# Endovascular Thrombectomy Versus Best Medical Management in Patients With Large Vessel Occlusion Stroke Presenting Beyond 24 Hours: Results From the TRACK‐LVO Late Multicenter Cohort

**DOI:** 10.1161/SVIN.124.001609

**Published:** 2025-02-11

**Authors:** Yongbo Xu, Shuling Liu, Adnan I. Qureshi, Pinyuan Zhang, Xiaochen Zhang, Shuai Liu, Yuanyuan Xue, Fanlei Meng, Guodong Xu, Yongchang Liu, Youquan Gu, Yibin Cao, Yanzhao Xie, Zhen Hong, Wanchao Shi, Yan Wang, Huisheng Chen, Ming Wei

**Affiliations:** ^1^ Clinical College of Neurology, Neurosurgery and Neurorehabilitation Tianjin Medical University Tianjin China; ^2^ Department of Neurosurgery Tianjin Huanhu Hospital Tianjin China; ^3^ Zeenat Qureshi Stroke Institute and Department of Neurology University of Missouri Columbia MO; ^4^ Department of Neurosurgery (Cerebrovascular Disease) The Third Hospital of Hebei Medical University Shijiazhuang China; ^5^ Department of Radiology Tianjin Huanhu Hospital Tianjin China; ^6^ Department of Neurosurgery The Second Hospital of Tianjin Medical University Tianjin China; ^7^ Department of Neurology Hebei General Hospital Shijiazhuang Hebei China; ^8^ Department of Neurovascular Intervention Cangzhou Central Hospital Cangzhou Hebei China; ^9^ Department of Neurology First Hospital of Lanzhou University Lanzhou China; ^10^ Department of Neurology Tangahan Gongren Hospital Tangshan City Hebei China; ^11^ Department of Neurosurgery Peking University BinHai Hospital Tianjin China; ^12^ Department of Neurology General Hospital of Northern Theater Command Shenyang China; ^13^ Department of Academy of Medical Engineering and Translational Medicine Tianjin University Tianjin China

**Keywords:** ischemic stroke, late treatment, thrombectomy

## Abstract

**BACKGROUND:**

The efficacy and safety of endovascular thrombectomy (EVT) performed beyond 24 hours from the last known well remain uncertain. This study aims to investigate the potential benefits of EVT versus best medical management (BMM) beyond 24 hours.

**METHODS:**

TRACK‐LVO Late (Late Triage of Patients Presenting Beyond 24 Hours With Acute Ischemic Stroke Due to Large Vessel Occlusions) is an ongoing, multicenter, prospective cohort study. A total of 410 individuals met the inclusion and exclusion criteria and were included in the cohort analyses from 2018 to 2024. The primary outcome was functional independence, defined as a modified Rankin Scale score of 0–2 at 90 days. Safety outcomes included all‐cause mortality within 90 days and symptomatic intracranial hemorrhage. A propensity score analysis was conducted to adjust for baseline imbalances. The association between treatment and primary outcome/safety outcomes was assessed using logistic regression, adjusted for age, sex, National Institutes of Health Stroke Scale score, premorbid modified Rankin Scale score, occlusion sites, and time from onset to admission.

**RESULTS:**

Among the 410 patients, 209 were in the EVT group and 201 in the BMM group. The EVT group showed higher odds of functional independence in the propensity score‐matched cohort (adjusted odds ratio, 4.13 [95% CI, 2.42–7.05]; *P*<0.001). No significant difference in mortality rate was observed between groups (adjusted odds ratio, 1.59 [95% CI, 0.60–4.25]; *P* = 0.354). However, the EVT group had an increased risk of symptomatic intracranial hemorrhage compared with the BMM group (adjusted odds ratio, 8.72 [95% CI, 1.04–73.10]; *P* = 0.046). These findings were consistent in sensitivity analyses using propensity score inverse probability of treatment weighting.

**CONCLUSION:**

EVT performed after 24 hours from the last known well was associated with higher rates of functional independence compared with BMM and demonstrated acceptable safety. High‐quality randomized trials are needed to further compare EVT and BMM beyond 24 hours from the last known well.

## Graphical abstract

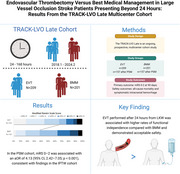

Acute ischemic stroke (AIS) caused by large vessel occlusion (LVO) is a significant global health concern, leading to substantial disability and mortality. The establishment of current guidelines for thrombolysis and thrombectomy has ushered in the era of reperfusion therapy for acute LVO. The DAWN (Diffusion Weighted Imaging or Computerized Tomography Perfusion Assessment With Clinical Mismatch in the Triage of Wake Up and Late Presenting Strokes Undergoing Neurointervention) and DEFUSE 3 (Endovascular Therapy Following Imaging Evaluation for Ischemic Stroke 3) randomized controlled trials have validated the safety and efficacy of endovascular thrombectomy (EVT) within a 24‐hour window for AIS due to LVO.^1^, ^2^ However, a large proportion of patients who present beyond 24 hours are excluded based on these criteria.

Stroke severity, extent, and progression vary widely, and earlier studies have demonstrated that salvageable brain tissue may persist beyond 24 hours in both preclinical and clinical studies. Patients with salvageable brain tissue may still benefit from EVT. Despite this, only a limited number of studies with modest sample sizes have compared the outcomes of EVT with best medical management (BMM) in this very late time window.^3^, ^4^, ^5^, ^6^, ^7^, ^8^, ^9^ A significant number of patients with LVO who present beyond 24 hours continue to experience severe disability due to the lack of evidence supporting interventions to recanalize occluded vessels.^6^, ^8^, ^10^

This study explored the therapeutic benefit of EVT for patients presenting beyond 24 hours from symptom onset or last known well (LKW). Using data from the TRACK‐LVO Late (Late Triage of Patients Presenting Beyond 24 Hours With Acute Ischemic Stroke Due to Large Vessel Occlusions) cohort, we assessed efficacy and safety outcomes in patients treated with EVT compared to those receiving BMM.

## METHODS

### Ethical Considerations

The data that support the findings of this study are available from the corresponding author upon reasonable request. The study was approved by the institutional review boards at each participating institution. All patients or their legally authorized representatives provided signed informed consent. The TRACK‐LVO Late study is registered on ClinicalTrial.gov (NCT06200753).

### Study Design

The TRACK‐LVO Late is an ongoing, prospective, multicenter cohort study linked to the TRACK‐LVO registry, enrolling patients from 9 high‐volume certified stroke centers in China, all of which also participate in the TRACK‐LVO Registry (NCT05659160). This cohort aims to evaluate the real‐world application of EVT and BMM in consecutive patients diagnosed with AIS caused by LVO in the anterior circulation, presenting beyond 24 hours from LKW.

Deidentified data on epidemiological, demographic, clinical, therapeutic, and outcome measures are managed via a web‐based interface. Data collection begins upon admission to the designated stroke unit, with subsequent entry of collected data throughout hospitalization until discharge. Follow‐up extends to 90 days post discharge. Standardized definitions for all variables and scores are rigorously applied to ensure consistency and accuracy in data collection.

### Participant Selection

This cohort enrolled consecutive patients meeting the eligibility criteria between January 1, 2018, and February 29, 2024. Inclusion criteria included (1) AIS symptoms manifesting 24 to 168 hours from symptom onset or LKW, and (2) LVO in the anterior circulation, including the intracranial internal carotid artery and/or the middle cerebral artery (M1 or M2 segments). Exclusion criteria were (1) transient ischemic attack at admission, (2) terminal medical conditions (eg, stage IV cancer), and (3) any form of cerebral hemorrhage diagnosed at admission.

**Nonstandard Abbreviations and Acronyms**

AIS: acute ischemic stroke
BMM: best medical management
EVT: endovascular thrombectomy
ICAD: intracranial atherosclerotic disease
LKW: last known well
LVO: large vessel occlusion
mRS: modified Rankin Scale
NIHSS: National Institutes of Health Stroke Scale
sICH: symptomatic intracranial hemorrhage
TRACK‐LVO Late: Late Triage of Patients Presenting Beyond 24 Hours With Acute Ischemic Stroke Due to Large Vessel Occlusions

**CLINICAL PERSPECTIVE**

**What Is New?**

- This study demonstrates that endovascular thrombectomy may be effective for patients with acute ischemic stroke presenting beyond 24 hours from last known well, a time window that has not been thoroughly investigated in previous randomized controlled trials.

**What Are the Clinical Implications?**

- Expanding the therapeutic window for endovascular thrombectomy could provide new treatment opportunities for a substantial population of patients with stroke who currently have limited therapeutic options due to late presentation.

### Study Treatments and Interventions

Patients with acute stroke were evaluated for eligibility before inclusion in the cohort. Treatment was based on patient preferences and physician recommendations, provided by a multidisciplinary stroke care team. Patients received either primary EVT combined with BMM or BMM alone. EVT included stent retrievers, aspiration, balloon angioplasty, stent implantation, or combinations of these techniques. The use of intra‐arterial thrombolytics was at the discretion of the neurointerventionalist. All patients received BMM per local protocol or Chinese guidelines for the diagnosis and treatment of AIS.

### Baseline Demographic and Clinical Assessments

Demographic and clinical assessments included age, sex, initial National Institutes of Health Stroke Scale (NIHSS) score at admission, premorbid modified Rankin Scale (mRS) score, Alberta Stroke Program Early Computed Tomography Score, ischemic core volume, and perfusion parameters (eg, mismatch volume, Tmax >6/8/10) when computed tomography (CT)/magnetic resonance perfusion imaging examination was available. Other variables assessed included LVO location (internal carotid artery, M1/M2 segment of the middle cerebral artery, and/or tandem lesion), time from LKW to admission, cardiovascular risk factors, comorbidities, and anesthetic modality (local or general anesthesia). Thrombolysis with alteplase was administered to patients within a 4.5‐hour window when indicated. Angiographic recanalization was documented using the modified Treatment in Cerebral Infarction score.

### Outcome Measures

The primary outcome was functional independence, defined as an mRS score of 0–2 at 90 days, assessed during follow‐up consultation or via structured telephonic interviews conducted by trained research nurses if in‐person visit were not feasible. Safety outcomes included symptomatic intracranial hemorrhage (sICH), defined as any hemorrhagic transformation on follow‐up imaging associated with a ≥4‐point increase in NIHSS score during hospitalization,^11^ and all‐cause mortality within the 90‐day follow‐up period.

### Statistical Analysis 

We compared demographics, baseline clinical and imaging characteristics, as well as outcomes between the 2 treatment groups. Continuous variables are presented as median with interquartile ranges, and categorical variables are expressed as counts and percentages. Univariate comparisons of categorical variables were performed using the chi‐square test or Fisher's exact test, as appropriate. Continuous variables were analyzed using the unpaired *t* test or the Mann–Whitney *U* test, depending on the distribution, which was assessed using the Shapiro–Wilk test.

To adjust for baseline differences between the treatment groups, propensity score (PS) analysis was conducted. The propensity score matching (PSM) population was generated using the nearest neighbor method within a caliper of 0.2 SDs of the logit of PS, without replacement, in a 1:1 ratio. For the PS weighting population, the stabilized average treatment effect weighting method was employed. Patients receiving EVT were assigned a weight of (proportion of patients receiving EVT)/(PS), whereas those receiving BMM were assigned a weight of (1‐proportion of patients receiving BMM)/(1‐PS). To enhance equipoise and minimize bias, the tails of the PS distributions were trimmed by excluding observations at or below the first percentile for EVT and at or above the 99th percentile for BMM. The effectiveness of inverse probability treatment weighting (IPTW) and PSM was assessed by calculating standardized mean differences for the baseline characteristics, with successful balance defined as standardized mean differences <10%.

Following PS adjustment, the association between the treatment group and outcomes—functional independence at 90 days, mortality within 90 days, and sICH during hospitalization—was analyzed using a multivariable logistic regression model. The main analysis was conducted in the PSM populations, whereas the IPTW populations were used for sensitivity analysis. Because the IPTW populations include multiple data points from individual patients, we computed a robust variance‐covariance of the model parameters to account for intrapatient correlations.

We also examined the heterogeneity of treatment effect size for favorable functional outcomes across subgroups defined by age (<65 versus ≥65 years), sex (male versus female), occlusion site (M1 segment, M2 segment, distal internal carotid artery, and proximal internal carotid artery), baseline NIHSS score (<6 versus ≥6), time from stroke onset to arrival (24–72, 72–120, and 120–168 hours), prestroke mRS score (0, 1, and ≥2), intravenous thrombolysis (yes versus no), history of atrial fibrillation (AF) (yes versus no). Multiplicative terms were included in the regression models to evaluate the statistical significance of interaction with treatment assignments.

Statistical significance was set at *P*<0.05. All analyses were performed using R version 4.3.2.

## RESULT

### Baseline Characteristics

This study included a final population of 410 consecutive patients with AIS from the TRACK‐LVO Late Cohort (flow chart in Figure S1). The proportions of patients with diabetes (EVT 23.9% versus BMM 38.3%; *P* = 0.002) and history of previous stroke (EVT 27.3% versus BMM 42.8%; *P* = 0.001) was higher in the BMM group. EVT patients were less likely to consume alcohol (EVT 28.3% versus BMM 41.0%; *P* = 0.008) or smoke cigarettes (EVT 36.4% versus BMM 51.0%; *P* = 0.003). Table 1 provides a summary of the clinical, imaging, and procedural characteristics.

**Table 1:** Baseline Information of the Entire Study Population

	No./total No.(%)		
	EVT group	BMM group	*P* value
No. of patients	209	201	
**Demographics**			
Age, y, mean±SD	63.00 (56.0–70.0)	65.00 (57.0–71.0)	0.061
**Sex**			
Female	52 (24.9)	59 (29.4)	0.308
Male	157 (75.1)	142 (70.6)	
**Cardiovascular risk factors**			
Hypertension	137 (65.6)	148 (73.6)	0.076
Diabetes	50 (23.9)	77 (38.3)	0.002
Previous stroke	57 (27.3)	86 (42.8)	0.001
Atrial fibrillation	18/198 (8.6)	10/201 (5.0)	0.172
Coronary artery disease	25 (12.6)	38 (18.9)	0.082
Cigarette smoking	72 (36.4)	102 (51.0)	0.003
Alcohol consumption	56 (28.3)	82 (41.0)	0.008
Antiplatelets	36 (23.4)	62 (30.8)	0.119
Anticoagulant	5 (3.2)	8 (4.0)	0.715
Intravenous thrombolysis	18 (9.0)	16 (8.0)	0.858
NIHSS score at admission, median (IQR)	10.0 (6.0–14.0)	9.0 (5.0–14.0)	0.055
**Transfer status**			
Direct admission	96 (52.7)	110 (54.7)	0.698
Transfer from another hospital	86 (47.3)	91 (45.3)	
Time from LKW to arrival of treating hospital, median (IQR), h	35.0 (27.5–52.0)	42.0 (28.0–68.0)	0.140
**Presentation of stroke**			
Wake up	40 (22.0)	36 (17.9)	0.560
Unwitnessed	47 (25.8)	51 (25.4)	
Witnessed	95 (52.2)	114 (56.7)	
**Premorbid mRS score**			
0	180 (89.6)	173 (86.1)	0.128
1	15 (7.5)	22 (10.9)	
2	1 (0.5)	5 (2.5)	
3	3 (1.5)	1 (0.5)	
4	2 (1.0)	0 (0.0)	
**Occlusion site**			
M1	114 (54.5)	98 (48.8)	0.687
M2	13 (6.2)	14 (7.0)	
ICA	67 (32.1)	71 (35.3)	
Tandem	15 (7.2)	18 (9.0)	
ASPECTS, median (IQR)	8.0 (7.0–9.0)	8.00 (7.0–9.0)	0.263
Ischemic core volume, median (IQR), mL	8.1 (2.97–17.20)	4.05 (0.00–17.42)	0.058
Mismatch volume (IQR), mL	69.95 (47.55–104.82)	109.1 (75.23–119.7)	0.088
Tmax>6 seconds (IQR), mL	85.5 (60.75–106.68)	127.4 (92.5–207.23)	0.042
Tmax>8 seconds (IQR), mL	32.0 (22.0–61.0)	76.85 (13.35–136.3)	0.321
Tmax>10 seconds (IQR), mL	16.0( 5.0–34.0)	39.45 (2.22–89.05)	0.352
**Anesthesia**			
Local	74 (45.7)	NA	NA
General	88 (54.3)	NA	
**Final mTICI**			
0	8 (4.3)	NA	NA
1	3 (1.6)	NA	
2a	9 (4.8)	NA	
2b	35 (18.7)	NA	
3	132 (70.6)	NA	
Diastolic BP^*^, median (IQR), mmHg	80.0 (76.8–91.3)	85.0 (74.6–94.3)	0.225
Systolic BP^*^, median (IQR), mmHg	145.0 (130.0–156.3)	149.5 (138.0–160.0)	0.078

ASPECTS indicates Alberta Stroke Program Early Computed Tomography Score; BMM, best medical treatment; BP, blood pressure; EVT, endovascular therapy; IQR, interquartile ranges; LKW, last known well; mRS, modified Rankin Scale; mTICI, modified thrombolysis in cerebral infarction; NA, not applicable; and NIHSS, National Institutes of Health Stroke Scale.

* BP was recorded when patients returned to the ward.

### Clinical Outcome Comparison After PSM

**Figure 1:**
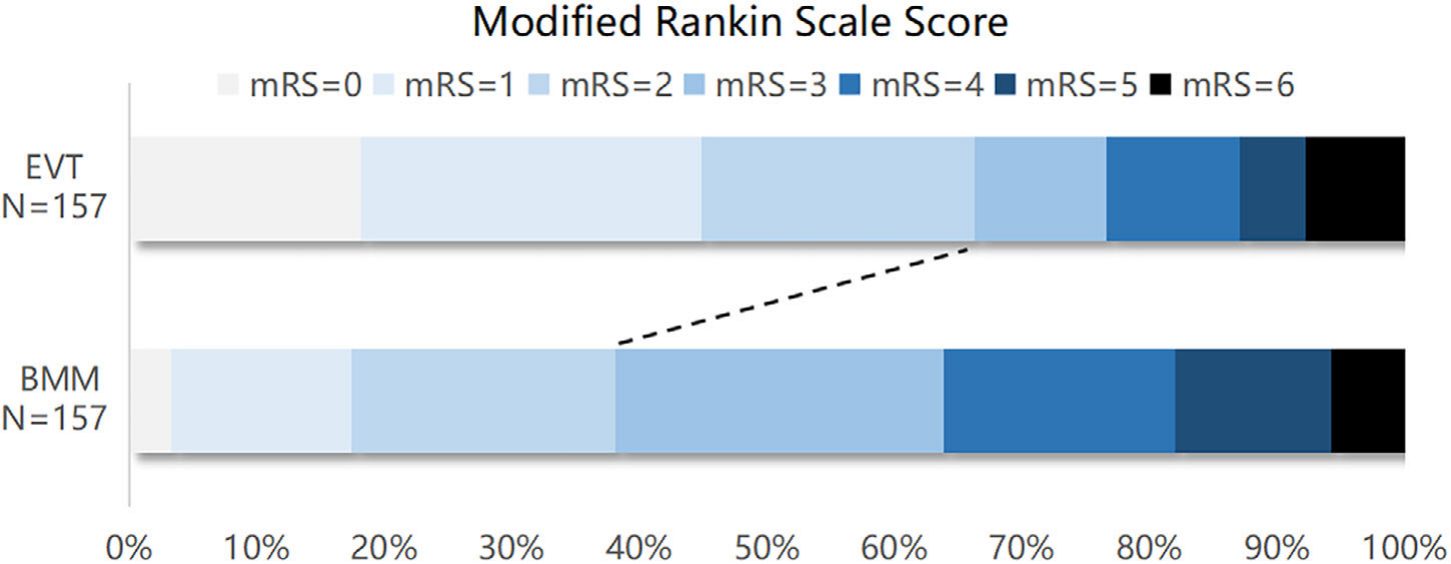
**Distribution of mRS scores at 90 days in propensity score matching population**. BMM, best medical management; EVT, endovascular thrombectomy; and mRS, modified Rankin Scale.

Following PSM, 157 matched pairs were identified, with baseline information details in Table S1. A significantly higher proportion of functional independence was observed in the EVT group compared with the BMM group (66.2% versus 38.1%, *P*<0.001). The adjusted odds ratios (aOR) for 90‐day functional independence was significantly higher for EVT group (aOR, 4.13 [95% CI, 2.42–7.05]; *P*<0.001) (see Figure 1 for the distribution). The benefits of EVT remained consistent across all time points from LKW to admission (Figure S2).

There was no significant difference in all‐cause mortality within 90 days between the 2 groups (EVT 7.8% versus BMM 5.8%; aOR, 1.59 [95% CI, 0.60–4.25]; *P* = 0.354). However, the occurrence of sICH was higher in the EVT group (5.1% versus 0.6% for control; aOR, 8.72 [95% CI, 1.04–73.10]; *P* = 0.046). These findings were consistent with the efficacy and safety outcomes observed in the entire population (Table 2).

**Table 2:** Efficacy and Safety Outcomes of the Entire Study Population

	No./total No.(%)		
	EVT group	BMM group	*P* value
No. of patients	209	201	
mRS score 0–1	81 (40.1)	37 (18.6)	<0.001
mRS score 0–2	121 (59.9)	77 (38.7)	<0.001
mRS score 0–3	147 (72.8)	124 (62.3)	0.025
mRS score 5–6	30 (14.9)	38 (19.1)	0.257
All‐cause mortality	18 (8.9)	14 (7.0)	0.488
sICH	12 (5.8)	1 (0.5)	0.002
Any intracranial hemorrhage	26 (12.5)	10 (5.0)	0.008

BMM indicates best medical treatment; EVT, endovascular thrombectomy; mRS, modified Rankin Scale; and sICH, symptomatic intracranial hemorrhage.

### Clinical Outcome Comparison After Inverse Probability of Treatment Weighting

Baseline information for IPTW population is shown in Table S2. The EVT group had a higher proportion of functional independence at 90 days in the EVT group (EVT: 65.0% versus BMM: 38.5%), with significantly higher aOR for 90‐day functional independence (aOR, 3.66 [95% CI, 2.27–5.90]; *P*<0.001). The all‐cause mortality rate within 90 days was similar between the 2 groups (EVT 7.9% versus BMM 6.7%; aOR, 1.38 [95% CI, 0.59–3.23]; *P* = 0.463). However, the risk of sICH was higher in the EVT group (5.8% versus 0.8% for BMM; aOR, 9.17 [95% CI, 1.51–55.59]; *P* = 0.016).

### Clinical Outcomes of Directly Admitted Patients

Among 206 patients directly admitted to EVT‐capable centers, 96 underwent EVT, whereas 110 received BMM. EVT was significantly associated with a higher likelihood of achieving functional independence at 90 days compared with BMM (68.8% versus 38.9%; OR, 6.88 [95% CI, 3.22–14.70]; *P*<0.001). No significant difference was observed in all‐cause mortality within 90 days between the 2 groups (EVT: 8.6% versus BMM: 7.4%; aOR, 0.86 [95% CI: 0.27–2.78]; *P* = 0.805). However, the incidence of sICH was higher in the EVT group (7.4% versus 0.0% in the control group).

### Subgroup Analysis

**Figure 2:**
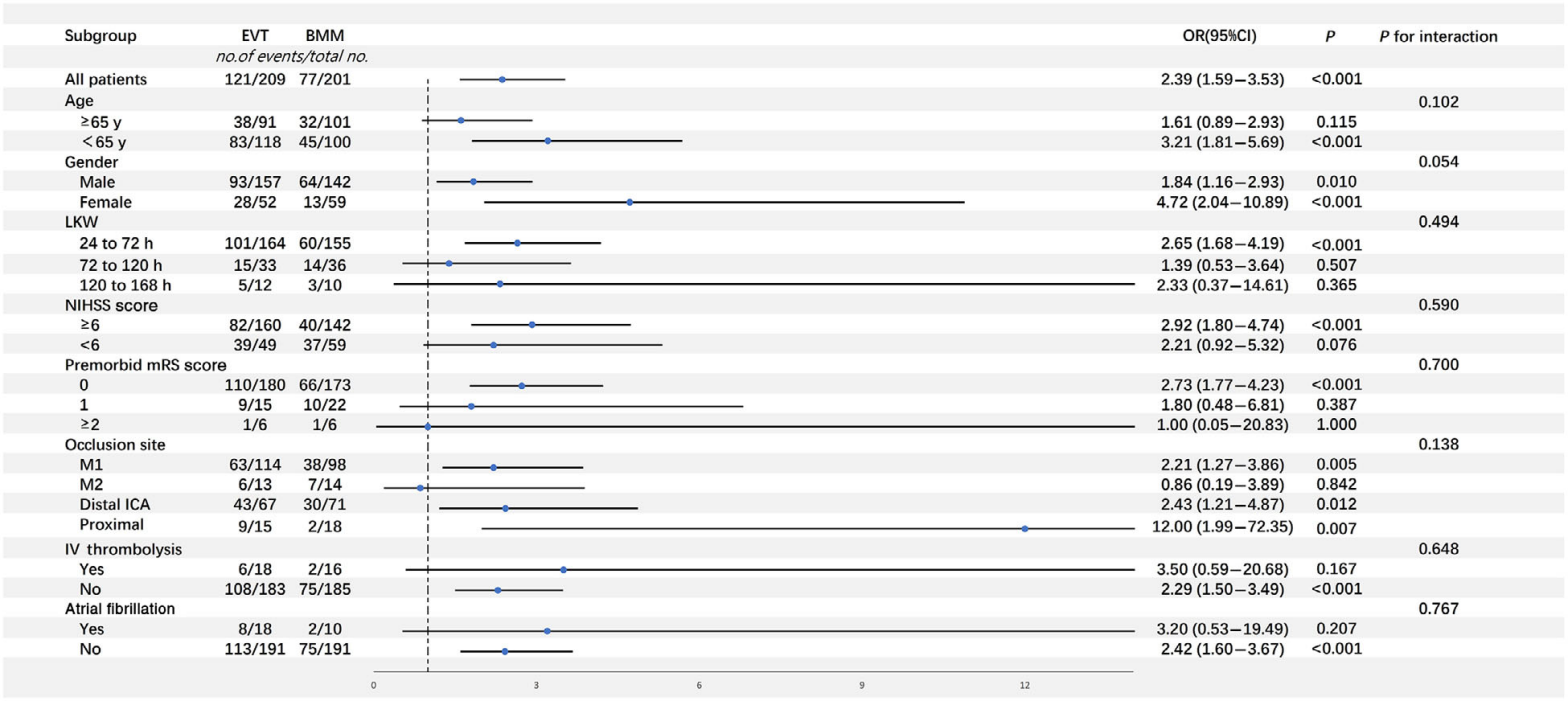
**Benefit of EVT versus BMM in achieving functional independence in different prespecified subgroup**. BMM indicates best medical management; EVT, endovascular thrombectomy; ICA, internal carotid artery; IV, intravenous; LKW, last known well; M1, segment 1 of middle cerebral artery; M2, segment 2 of middle cerebral artery; mRS, modified Rankin Scale; NIHSS, National Institutes of Health Stroke Scale; and OR, odds ratio.

The association between achieving functional independence at 90 days and EVT was consistent across all subgroups, with no significant interaction observed (Figure 2).

### Additional Analysis in Population With Retrospective Volumetric Analysis

#### Association of Functional Independence With Baseline Ischemic Core Volume

**Figure 3:**
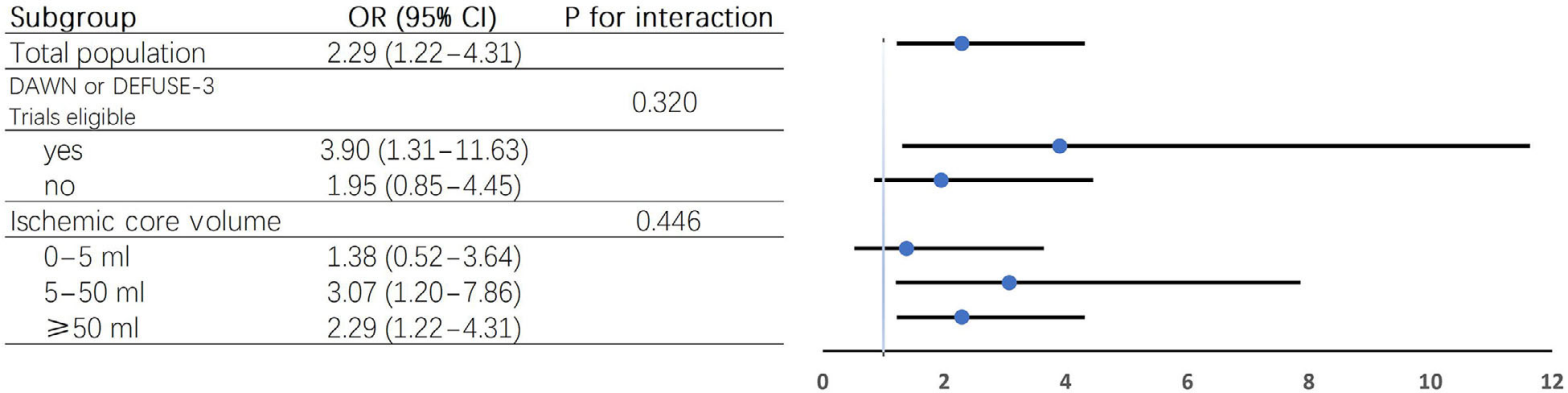
**Subgroup analysis of functional independence in patients who received advanced imaging**. DAWN indicates Diffusion Weighted Imaging or Computerized Tomography Perfusion Assessment With Clinical Mismatch in the Triage of Wake Up and Late Presenting Strokes Undergoing Neurointervention; DEFUSE 3, Endovascular Therapy Following Imaging Evaluation for Ischemic Stroke 3; and OR, odds ratio.

Among the total population, retrospective analysis of ischemic core volume was conducted on 163 patients using F‐stroke (Neuroblem V1.0.19). Figure S3 illustrates that the likelihood of achieving functional independence decreases as the ischemic core volume increases in both EVT and BMM groups. Patients receiving EVT had a generally higher probability of functional independence compared with those receiving BMM. Subgroup analysis revealed that patients with a baseline ischemic core volume of 5–50 mL were more likely to achieve functional independence than those with core volumes either <5 mL or >50 mL (Figure 3).

#### DAWN/DEFUSE 3 Eligible Versus DAWN/DEFUSE 3 Ineligible Subgroups

Perfusion imaging data were available for 32 patients. 70 patients met DAWN/DEFUSE 3 eligibility criteria, whereas the remaining 93 were ineligible. Table S3 and Table S4 summarize the baseline characteristics of these groups.

- In the DAWN/DEFUSE 3 eligible subgroup, EVT was associated with significantly higher rates of functional independence at 90 days (EVT: 47.4% versus BMM: 18.8%; OR, 3.90 [95% CI, 1.31–11.63]; *P* = 0.015) (Figure 3).
- In the DAWN/DEFUSE 3 ineligible subgroup, EVT was not associated with significantly higher rates of functional independence (EVT: 60.5% versus BMM: 44.0%; OR, 1.95 [95% CI, 0.85–4.45]; *P* = 0.115) (Figure 3). Exploratory analysis excluding patients who met DAWN or DEFUSE 3 criteria but had admission NIHSS scores <10 or 6, respectively, showed an increased point estimate for functional independence, rising from 1.95 to 4.28.

#### Comparison of DAWN Versus DEFUSE 3 Criteria

**Figure 4:**
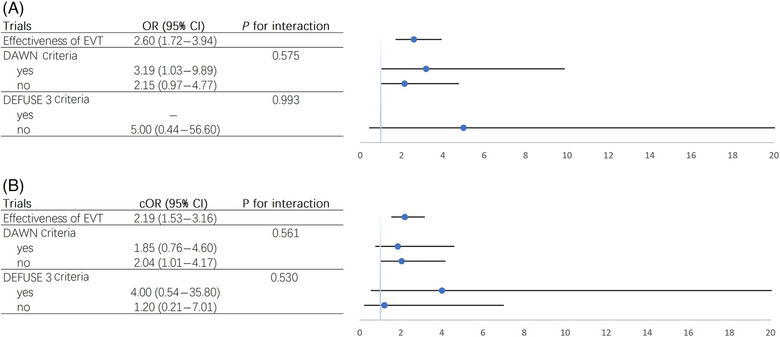
**Benefit of EVT in DAWN and DEFUSE 3 subgroups**. **A**, Binary logistic regression to calculate the OR for achieving an mRS score of 0–2; (**B**) Ordered logistic regression to calculate the cOR for the trend of improvement in mRS scores. Due to the complete separation phenomenon, OR calculation is infeasible. cOR indicates crude odds ratio; DAWN, Diffusion Weighted Imaging or Computerized Tomography Perfusion Assessment With Clinical Mismatch in the Triage of Wake Up and Late Presenting Strokes Undergoing Neurointervention; DEFUSE 3, Endovascular Therapy Following Imaging Evaluation for Ischemic Stroke 3; EVT, endovascular thrombectomy; mRS, modified Rankin Scale; and OR, odds ratio.

A total of 63 patients met only the DAWN criteria, whereas 15 met only the DEFUSE 3 criteria. The imaging criteria from both trials did not significantly alter the effectiveness of EVT in achieving an mRS score of 0–2 (Figure 4A) or in promoting a favorable shift in mRS scores (Figure 4B) for patients with LVO presenting beyond 24 hours. However, the DEFUSE 3 criteria demonstrated a slightly higher point estimate for functional recovery compared with the DAWN criteria.

## DISSCUSION

Our study shows that EVT performed beyond 24 hours from LKW is associated with higher rates of functional independence compared with BMM and with an acceptable safety profile. Notably, the benefit appeared most prominent among patients meeting the DAWN/DEFUSE 3 criteria. A substantial proportion of patients presented beyond 24 hours from the time of LKW or symptom onset, representing a significant demographic within the stroke population. Although salvageable ischemic penumbra may persist beyond 24 hours in some individuals, particularly among “slow progressors” characterized by a small infarct core and robust collateral supply,^12^, ^13^ these patients remain at risk of death or disability without reperfusion therapy, due to infarction of the penumbra tissue.^14^ However, there is uncertainty whether EVT can reduce death or disability in these patients, given the absence of supporting randomized controlled trials.^15^, ^16^

Several observational studies have reported outcomes of EVT beyond 24 hours, though few directly compared EVT and BMM. A retrospective single‐center study evaluated outcomes in 13 patients who received EVT and 96 who received BMM, all presenting beyond 24 hours. This study demonstrated a trend toward improved functional outcomes with EVT.^8^ Furthermore, a multicenter retrospective study of 185 patients receiving EVT and 116 with BMM suggested that EVT was associated with higher rates of functional independence (38% versus 10%; aOR, 4.56 [95% CI, 2.28–9.09]) in an IPTW population.^17^ Another single‐center retrospective study used non‐contrast CT/CT angiography to select eligible patients presenting beyond 24 hours for EVT, including 19 patients receiving EVT and 16 with BMM, and found higher rates of functional independence (36.9% versus 18.8%; aOR, 4.34 [95% CI, 0.34–54.83]). Our prospective cohort study provides additional data supporting that EVT is associated with higher odds of functional independence at 90 days compared with BMM (aOR, 4.13 [95% CI, 2.42–7.05]; *P*<0.001). Our prospective cohort is unique in its larger sample size, inclusion of an Asian population (where intracranial atherosclerotic disease [ICAD] is prevalent), and reliance on magnetic resonance imaging in over half of the cases. Unlike many late‐window trials, we did not exclude patients with minor strokes (NIHSS score <6), as they represent a substantial proportion of EVT candidates in clinical practice. These design elements allow our findings to offer practical insights into patient selection and potential treatment benefits.

A quarter of the patients in this study presented with a mild stroke. Prior research comparing EVT and BMM did not demonstrate EVT's superiority in achieving functional independence.^18^ In our study, we observed better outcome in achieving functional independence for patients with minor stroke presenting beyond 24 hours (OR, 2.21 [95% CI, 0.92–5.32]; *P* = 0.076), as further evidenced by a point estimate increase in proportional difference of 16.9%—a value exceeding those reported in earlier studies conducted within a 24‐hour window. Because prior data show that about one quarter of patients with AIS with LVO and mild deficits fail to achieve functional independence by 3 months when managed medically,^19^ the elevated rate of not achieving functional dependence in our BMM group (37.3%) may suggest a meaningful role for EVT in these patients with late‐presenting, minor stroke. Nonetheless, our sample was small, and further research is necessary to confirm these observations.

In our study, the lower prevalence of AF compared with the DAWN trial intervention arm (12% versus 40%) may be attributed to the higher incidence of ICAD in Asian populations, where ICAD is a more common cause of LVO and typically correlates with lower rates of AF. Additionally, as noted earlier, patients presenting beyond 24 hours had relatively low NIHSS scores, which may also contribute to the lower prevalence of AF. A recent study focusing on Asian patients presenting beyond 24 hours with mild symptoms reported an AF prevalence of 15%–17%, aligning with our findings.^20^ These observations highlight the importance of considering population‐specific factors, such as the prevalence of ICAD and symptom severity, when interpreting the results and assessing the generalizability of our study to other populations.

Current guidelines recommend selecting patients for EVT between 6 and 24 hours based on stringent clinical and imaging criteria (DEFUSE 3 or DAWN).^15^ Recent evidence even suggested that selection based on non‐contrast CT scans may be adequate for selecting patients with AIS presenting within 24 hours.^21^ Yet, there is a paucity of evidence regarding the appropriate selection criteria for patients presenting beyond 24 hours. Prior studies often selected these patients using advanced neuroimaging (CT perfusion or magnetic resonance imaging) similar to the inclusion criteria of late window trials (6–24 hours).^5^, ^8^, ^17^ However, an optimal approach to identifying patients who may benefit from EVT remains uncertain. In our study, patients deemed eligible by DAWN/DEFUSE 3 criteria fared significantly better with EVT, whereas those outside these criteria did not show a clear benefit, although the point estimate for functional independence rose when further exploratory exclusions were made. The lack of a statistically significant difference might be attributed to the relatively small sample size, potentially leading to an underpowered result.

Only 44% of our participants underwent advanced imaging suitable for retrospective volumetric analysis, reflecting real‐world barriers such as inter‐hospital transfers, resource limitations, and physician preference. A single‐center study employed non‐contrast CT for estimated ischemic volume and CT angiography for collateral status to select eligible patients presenting beyond 24 hours from LKW.^6^ This approach was associated with a numerically but not statistically significant higher rate of functional independence in the EVT group compared with the medically managed group, thus its performance awaits confirmation in future large‐scale prospective trials. Interestingly, patients without advanced imaging still benefited from EVT in our study. This suggests that in practical scenarios, simpler imaging protocols may suffice for certain patients presenting beyond 24 hours, an idea warranting further large‐scale research.

The incidence of sICH (5.8%) in the cohort aligns with previous studies, which report rates of 4.8%–10.1%.^3^, ^4^, ^5^, ^6^, ^8^, ^17^ However, there was a striking difference in sICH rates when comparing the EVT and medical management group (EVT 5.8% versus BMM 0.5%; *P* = 0.003). The BMM cohort showed an exceptionally lower incidence of sICH, possibly due to progressive clot stabilization over time. By contrast, thrombectomy can disrupt the clot, induce reperfusion injury, and more easily cause intimal injury, thus raising the likelihood of sICH. Conversely, patients managed conservatively generally avoid clot disruption and reperfusion risks and thus typically have lower rate of sICH.

### Limitations

First, the observational nature of TRACK‐LVO Late may limit our ability to account for hidden biases from unmeasured risk factors. Addressing this rigorously would require a randomized controlled trial. Second, clinicians' decisions regarding EVT may have been influenced by factors not captured in our data. Third, despite PS adjustment, unidentified baseline imbalances may persist. Fourth, the study lacked access to all imaging data due to secondary patient transfers, missing imaging, and resource constraints preventing repeated imaging. Fifth, the absence of standardized guidelines and a uniform imaging protocol beyond 24 hours meant patient selection was largely based on institutional protocols or the treating physician's preference, limiting generalizability of our findings. Lastly, our findings' broader application may be constrained by the higher prevalence of ICAD in the Chinese population and a lower proportion of female participants.

## CONCLUSIONS

The therapeutic window for EVT could potentially be extended to improve proportion of patients with AIS achieving functional independence. For patients presenting beyond 24 hours, physicians should consider offering EVT over medical management.

## Author Contributions

Y. Xu, S. Liu, M. Wei, H. Chen contributed to the conception and design of the study. Y. Xu, S. Liu, P. Zhang, S. Liu, Y. Xue, X. Zhang, F. Meng, G. Xu, Y. Liu, Y. Gu, Y. Cao, Y. Xi, Z. Hong, W. Shi, Y. Wang, H. Che, and M. Wei contributed to the acquisition and analysis of data. Y. Xu, S. Liu, A. I. Qureshi, P. Zhang contributed to drafting the text or preparing the figures. M. Wei, H. Che, and A. I. Qureshi contributed to critical revisions of intellectual contents of the manuscript draft.

## Disclosures

A. I. Qureshi reports disclosures including consultancy relationships with Medtronic and Chiesi USA, as well as equity holdings in QuReVasc LLC and DyQure LLC; All other authors report no disclosures relevant to this study.

## Sources of Funding

National Health Commission Capacity Building and Continuing Education Center Nervous System and Minimally Invasive Intervention Program. No. GWJJ2022100106. Tianjin Health Science and Technology Project No. TJWJ2024ZD006. Beijing‐Tianjin‐Hebei Basic Research Cooperation Project (Grant number: 22JCZXJC00190). Tianjin Key Research and Development Program in Science and Technology, No. 19YFZCSY00260.

## Supporting information




**Figure S1**: Flow chart of current study.
**Figure S2**: Relationship between the delay from the time the patient was LKW (in hours) and the adjusted odds ratio (log‐transformed) for achieving functional independence at 3 months.
**Figure S3**: Association of ischemic core volume on admission and the probability of functional independence with EVT versus BMM in patients presenting beyond 24 hours.
**Table S1**: Baseline characteristics in PSM population.
**Table S2**: Baseline characteristics in IPTW population.
**Table S3**: Baseline characteristics in DDE population.
**Table S4**: Baseline characteristics in DDI population.
**Table S5**: Standardized mean difference before and after each propensity score analysis.
