# Supplementary material for: Endovascular Thrombectomy Versus Best Medical Management in Patients With Large Vessel Occlusion Stroke Presenting Beyond 24 Hours: Results From the TRACK‐LVO Late Multicenter Cohort
Source: Stroke Vasc Interv Neurol. 2025 Feb 11;5(2):e001609. doi: 10.1161/SVIN.124.001609 (PMC12671634; doi:10.1161/SVIN.124.001609)
Supplement: Supplementary file 1 — Figure S1: Flow chart of current study. Figure S2: Relationship between the delay from the time the patient was LKW (in hours) and the adjusted odds ratio (log‐transformed) for achieving functional independence at 3 months. Figure S3: Association of ischemic core volume on admission and the probability of functional independence with EVT versus BMM in patients presenting beyond 24 hours. Table S1: Baseline characteristics in PSM population. Table S2: Baseline characteristics in IPTW population. Table S3: Baseline characteristics in DDE population. Table S4: Baseline characteristics in DDI population. Table S5: Standardized mean difference before and after each propensity score analysis. [file SVI2-5-e001609-s001.docx]

**SUPPLEMENTAL MATERIALS**

**Endovascular Thrombectomy Versus Best Medical Management in Large Vessel Occlusion Stroke Patients Presenting Beyond 24 Hours: Results From the TRACK-LVO Late Multicenter Cohort**

**AUTHORS:**

Yongbo Xu^1^*, MD; Shuling Liu^1,2^*, PhD; Adnan I Qureshi^3^*, MD; Pinyuan Zhang^4^, MD; Xiaochen Zhang^5^, MD; Shuai Liu^1^, MD; Yuanyuan Xue^1^, MD; Fanlei Meng^6^, MD; Guodong Xu^7^, MD; Yongchang Liu^8^, MD; Youquan Gu^9^, MD; Yibin Cao^10^, MD; Yanzhao Xie^7^, MD; Zhen Hong^8^,MD; Wanchao Shi^11^, MD; Yan Wang^1^, MD; Huisheng Chen^12^, MD; and Ming Wei^1, 2, 13^, MD.

Corresponding Author(s):

Ming Wei, [drweiming@163.com](mailto:drweiming@163.com)

*Yongbo Xu, Shuling Liu and Adnan I Qureshi contributed equally to this article.

**AFFILIATIONS:**

1. Clinical College of Neurology, Neurosurgery and Neurorehabilitation, Tianjin Medical University, China.

2. Department of Neurosurgery, Tianjin Huanhu Hospital, Tianjin, China.

3. Zeenat Qureshi Stroke Institute and Department of Neurology, University of Missouri, Columbia, MO.

4. Department of Neurosurgery (Cerebrovascular Disease), The Third Hospital of Hebei Medical University, Shijiazhuang, China.

5. Department of Radiology, Tianjin Huanhu Hospital, Tianjin, China.

6. Department of Neurosurgery, The Second Hospital of Tianjin Medical University, Tianjin, China.

7. Department of Neurology, Hebei General Hospital, Shijiazhuang, Hebei, China

8. Department of Neurovascular Intervention, Cangzhou Central Hospital, Cangzhou, Hebei, China.

9. Department of Neurology, First Hospital of Lanzhou University, Lanzhou 730000, China.

10. Department of Neurology, Tangahan Gongren Hospital, Tangshan City, Hebei, China.

11. Department of Neurosurgery, Peking University BinHai Hospital, Tianjin, China.

12. Department of Neurology, General Hospital of Northern Theater Command, Shenyang, China.

13. Department of Academy of Medical Engineering and Translational Medicine, Tianjin University, Tianjin, China.

**Figure S1.** Flow chart of current study.

**Figure S2.** Relationship between the delay from the time the patient was LKW (in hours) and the adjusted odds ratio (log-transformed) for achieving functional independence at 3 months.

**Figure S3.** Association of ischemic core volume on admission and the probability of functional independence with EVT versus BMM in patients presenting beyond 24 hours.

**Table S1.** Baseline characteristics in PSM population.

**Table S2.** Baseline characteristics in IPTW population.

**Table S3.** Baseline characteristics in DDE population.

**Table S4.** Baseline characteristics in DDI population.

**Table S5.** Standardized mean difference before and after each propensity score analysis.

**Figure S1. Flow chart of current study**

**478** Patients with Large Vessel Occlusion in TRACK-LVO Late

Between 2018.1-2024.2

**410** Enrolled in Current Study

209 Received EVT

201 Received medical management

**77** Excluded:

72 Presenting beyond 7 days

3 Hemorrhage

2 Other reasons

**314** in PSM population

157 Received EVT

157 Received medical management

**381.8** in IPTW population

194.2 Received EVT

187.6 Received medical management

**163** in Population with Retrospective Volumetric Analysis

81 Received EVT

82 Received medical management

**Figure S2. Relationship between the delay from the time the patient was LKW (in hours) and the adjusted odds ratio (log-transformed) for achieving functional independence at 3 months.**


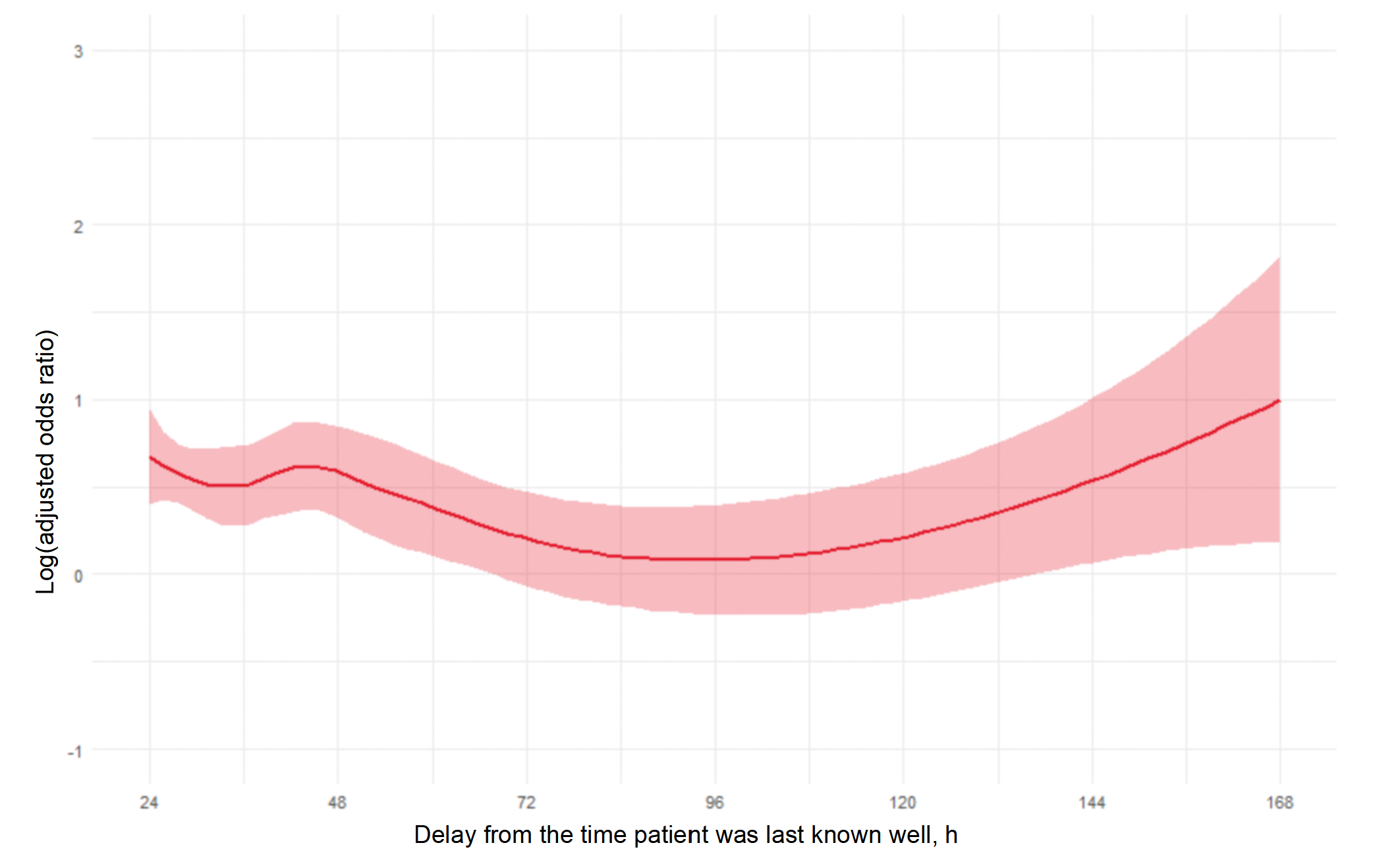


**Figure S3. Association of ischemic core volume on admission and the probability of functional independence with EVT versus BMM in patients presenting beyond 24 hours.**


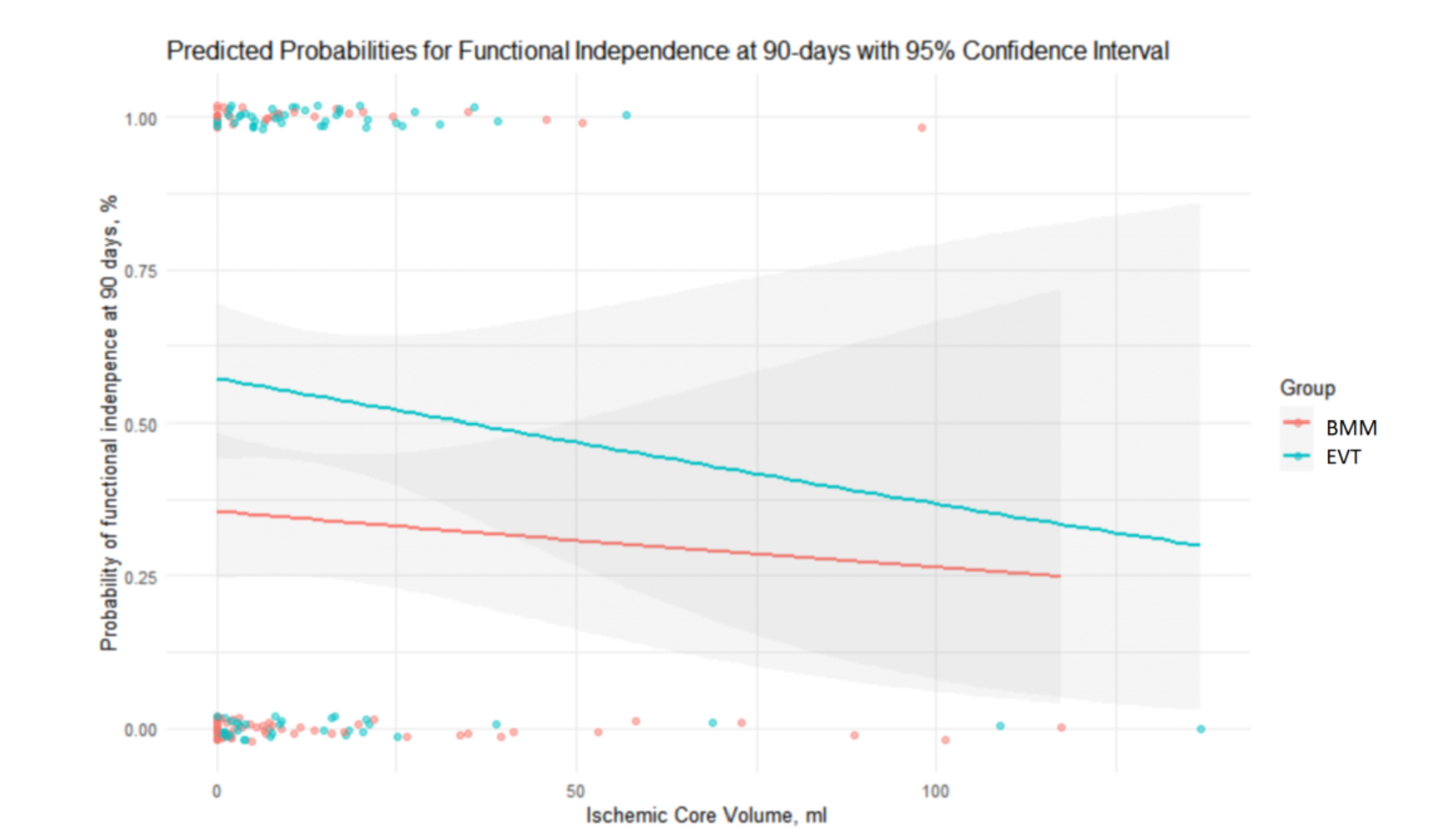


Abbreviation: EVT, Endovascular thrombectomy; BMM, Best medical management.

**Table S1. Baseline characteristics in PSM population**

|  | No./Total No.(%) |  |  |
| --- | --- | --- | --- |
|  | EVT group | BMM group | p.value |
| **No. of Patients** | 157 | 157 |  |
| **Demographics** |  |  |  |
| Age, median (IQR) | 63 (56-70) | 63 (55.70) | .651 |
| Gender |  |  |  |
| Female | 43 (27.4) | 44 (28.0) | 0.900 |
| Male | 114 (72.6) | 113 (72.0) |  |
| **Cardiovascular risk factors** |  |  |  |
| Hypertension | 109 (69.4) | 112 (71.3) | 0.710 |
| Diabetes mellitus | 43 (27.4) | 63 (40.1) | 0.017 |
| Previous stroke | 41 (26.1) | 66 (42.0) | 0.003 |
| Atrial Fibrillation | 15 ( 9.6) | 8 ( 5.1) | 0.125 |
| Coronary artery disease | 21 (14.2) | 27 (17.2) | 0.471 |
| Cigarette smoking | 56 (37.8) | 81 (51.9) | 0.014 |
| Alcohol consumption | 43 (29.1) | 67 (42.9) | 0.012 |
| Antiplatelets | 28 (23.1) | 46 (29.3) | 0.249 |
| Intravenous Thrombolysis | 13 ( 8.7) | 16 (10.2) | 0.662 |
| NIHSS score at admission, median (IQR) | 9.0 (5.0-13.0) | 9.0 (6.0-13.0) | .91 |
| Transfer status |  |  |  |
| Direct admission | 72 (52.2) | 82 (52.2) | 0.992 |
| Transfer from another hospital | 66 (47.8) | 75 (47.8) |  |
| Time from LKW to arrival of treating hospital, median (IQR), h | 34.0 (28.0-53.0) | 39.0 (27.0-66.0) | .001 |
| Premorbid mRS |  |  |  |
| 0 | 141 (89.8) | 141 (89.8) | 1 |
| 1 | 15 ( 9.6) | 14 ( 8.9) |  |
| 2 | 1 ( 0.6) | 1 ( 0.6) |  |
| 3 | 0 ( 0.0) | 1 ( 0.6) |  |
| 4 | 0 ( 0.0) | 0 ( 0.0) |  |
| Occlusion site |  |  |  |
| M1 | 81 (51.6) | 77 (49.0) | .946 |
| M2 | 11 ( 7.0) | 11 ( 7.0) |  |
| ICA | 52 (33.1) | 57 (36.3) |  |
| Tandem | 13 ( 8.3) | 12 ( 7.6) |  |
| ASPECT, median (IQR) | 8.0 (7.0-9.0) | 8.0 (7.0-9.0) | .119 |
| Ischemic Core Volume, median (IQR), mL | 7.6 (2.99-17.1) | 5.95 (0.15-18.0) | .256 |
| Mismatch Volume (IQR), ml | 70.8 (48.0- 105.8) | 110.1 (71.2-121.6) | .154 |
| Tmax>6s (IQR), ml | 85.0 (58.0-106.9) | 129.3 (87.3-215.3) | .054 |
| Tmax>8s (IQR), ml | 31.0 (18.9-59.7) | 77.4 (11.4-139.3) | .201 |
| Tmax>10s (IQR), ml | 13.1 (4.5-34.8) | 40.9 (4.1-103.9) | .2 |

Abbreviations: ASPECT: Alberta Stroke Program Early CT Score; BMM: Best Medical Management; EVT: Endovascular Thrombectomy; ICA: Internal Carotid Artery; IQR: Interquartile Range; LKW: Last Known Well; mRS: Modified Rankin Scale; M1: Middle Cerebral Artery, Segment 1; M2: Middle Cerebral Artery, Segment 2; NIHSS: National Institutes of Health Stroke Scale

**Table S2. Baseline characteristics in IPTW population**

|  | No./Total No.(%) |  |  |
| --- | --- | --- | --- |
|  | EVT group | BMM group | p.value |
| **No. of Patients** | 194.2 | 187.6 |  |
| **Demographics** |  |  |  |
| Age, median (IQR) | 64.0 (57.0-71.0) | 63.6 (56.0- 70.0) | .648 |
| Gender |  |  |  |
| Female | 52.0 (26.8) | 50.7 (27.0) | NA |
| Male | 142.3 (73.2) | 136.9 (73.0) |  |
| **Cardiovascular risk factors** |  |  |  |
| Hypertension | 129.4 (66.6) | 136.5 (72.8) | NA |
| Diabetes mellitus | 56.3 (29.0) | 69.9 (37.2) | NA |
| Previous stroke | 55.4 (28.5) | 78.5 (41.9) | NA |
| Atrial Fibrillation | 18.1 ( 9.3) | 10.3 ( 5.5) | NA |
| Coronary artery disease | 26.8 (14.5) | 32.8 (17.5) | NA |
| Cigarette smoking | 62.2 (34.0) | 98.8 (52.9) | NA |
| Alcohol consumption | 51.7 (28.3) | 81.6 (43.7) | NA |
| Antiplatelets | 37.4 (25.5) | 54.5 (29.1) | NA |
| Intravenous Thrombolysis | 16.3 ( 8.9) | 14.8 ( 7.9) | NA |
| NIHSS score at admission, median (IQR) | 9.0 (5.0-12.0) | 9.0 (5.0-12.0) | 0.671 |
| Transfer status |  |  |  |
| Direct admission | 90.5 (54.1) | 99.5 (53.0) | NA |
| Transfer from another hospital | 76.9 (45.9) | 88.2 (47.0) |  |
| Time from LKW to arrival of treating hospital, median (IQR), h | 36.0 (28.0-72.0) | 39.9 (27.0-66.8) | 0.981 |
| Premorbid mRS |  |  |  |
| 0 | 170.9 (88.0) | 166.6 (88.8) | NA |
| 1 | 20.1 (10.3) | 18.7 (10.0) |  |
| 2 | 2.5 ( 1.3) | 2.4 ( 1.3) |  |
| 3 | 0.8 ( 0.4) | 0.0 ( 0.0) |  |
| 4 | 0.0 ( 0.0) | 0.0 ( 0.0) |  |
| Occlusion site |  |  |  |
| M1 | 94.3 (48.6) | 96.3 (51.3) | NA |
| M2 | 13.4 ( 6.9) | 13.4 ( 7.1) |  |
| ICA | 71.5 (36.8) | 62.4 (33.2) |  |
| Tandem | 15.0 ( 7.7) | 15.6 ( 8.3) |  |
| ASPECT, median (IQR) | 8.0 (7.0-9.0) | 8.0 (7.0-9.0) | 0.365 |
| Ischemic Core Volume, median (IQR), mL | 6.9 (1.9-16.3) | 5.9 (0.2-20.1) | 0.636 |
| Mismatch Volume (IQR), ml | 69.9 (47.5-108.3) | 103.4 (68.0-112.3) | 0.189 |
| Tmax>6s (IQR), ml | 75.2 (55.7-114.8) | 123.0 (78.9- 201.0) | 0.043 |
| Tmax>8s (IQR), ml | 30.8(9.32-58.5) | 69.1 (9.81-138.0) | 0.252 |
| Tmax>10s (IQR), ml | 13.0 (2.6-34.5) | 35.7 (1.57-97.4) | 0.264 |

Abbreviations: ASPECT: Alberta Stroke Program Early CT Score; BMM: Best Medical Management; EVT: Endovascular Thrombectomy; ICA: Internal Carotid Artery; IQR: Interquartile Range; LKW: Last Known Well; mRS: Modified Rankin Scale; M1: Middle Cerebral Artery, Segment 1; M2: Middle Cerebral Artery, Segment 2; NIHSS: National Institutes of Health Stroke Scale

**Table S3. Baseline characteristics in DDE population**

|  | No./Total No.(%) |  |  |
| --- | --- | --- | --- |
|  | EVT group | BMM group | p.value |
| **No. of Patients** | 38 | 32 |  |
| **Demographics** |  |  |  |
| Age, mean (SD),y | 58.34 (13.12) | 63.38 (12.09) | .102 |
| Gender |  |  |  |
| Female | 8 (21.1) | 10 (31.2) | .331 |
| Male | 30 (78.9) | 22 (68.8) |  |
| **Cardiovascular risk factors** |  |  |  |
| Hypertension | 25 (65.8) | 23 (71.9) | .585 |
| Diabetes mellitus | 9 (23.7) | 14 (43.8) | .075 |
| Previous stroke | 7 (18.4) | 16 (50.0) | .005 |
| Atrial Fibrillation | 5 (13.2) | 1 (3.1) | .209 |
| Coronary artery disease | 3 (7.9) | 4 (12.5) | .695 |
| Cigarette smoking | 15 (39.5) | 15 (46.9) | .533 |
| Alcohol consumption | 7 (18.4) | 10 (31.2) | .212 |
| Antiplatelets | 4 (11.8) | 16 (50.0) | .001 |
| Intravenous Thrombolysis | 5 (13.9) | 5 (15.6) | .840 |
| NIHSS score at admission, median (IQR) | 12.0 (10.0- 13.75) | 12.0 (10.0- 14.0) | .752 |
| Transfer status |  |  |  |
| Direct admission | 16 (47.1) | 20 (62.5) | .208 |
| Transfer from another hospital | 18 (52.9) | 12 (37.5) |  |
| Time from LKW to arrival of treating hospital, median (IQR), h | 29.0 (25.62- 48.0) | 48.0 (29.0- 78.0) | .007 |
| Premorbid mRS |  |  |  |
| 0 | 33 (91.7) | 20 (62.5) | .007 |
| 1 | 3 (8.3) | 7 (21.9) |  |
| 2 | 0 (0.0) | 4 (12.5) |  |
| 3 | 0 (0.0) | 1 (3.1) |  |
| Occlusion site |  |  |  |
| M1 | 25 (65.8) | 9 (28.1) | <0.001 |
| M2 | 4 (10.5) | 0 (0.0) |  |
| ICA | 8 (21.1) | 19 (59.4) |  |
| Tandem | 1 (2.6) | 4 (12.5) |  |
| ASPECT, median (IQR) | 8.0 (8.0- 9.0) | 8.0 (7.0- 9.5) | .473 |
| Ischemic Core Volume, median (IQR), mL | 8.75 (3.15- 18.3) | 5.2 (1.05- 12.2) | .116 |
| Mismatch Volume (IQR), ml | 83.60 (64.07- 109.97) | 96.40 (70.23-246.17) | .544 |
| Tmax>6s (IQR), ml | 91.05 (77.70-112.15) | 127.40 (112.83-253.35) | .225 |
| Tmax>8s (IQR), ml | 32.50 (27.25- 59.70) | 76.85 (60.07-176.65) | .234 |
| Tmax>10s (IQR), ml | 14.05 (8.75- 34.77) | 39.45 (29.52-104.62) | .234 |

### Abbreviations: ASPECT: Alberta Stroke Program Early CT Score; BMM: Best Medical Management; DDE: DAWN or DEFUSE 3 Eligible; EVT: Endovascular Thrombectomy; ICA: Internal Carotid Artery; IQR: Interquartile Range; LKW: Last Known Well; mRS: Modified Rankin Scale; M1: Middle Cerebral Artery, Segment 1; M2: Middle Cerebral Artery, Segment 2; NIHSS: National Institutes of Health Stroke Scale; SD: Standard Deviation.

**Table S4. Baseline characteristics in DDI population**

|  | No./Total No.(%) |  |  |
| --- | --- | --- | --- |
|  | EVT group | BMM group | p.value |
| **No. of Patients** | 43 | 50 |  |
| **Demographics** |  |  |  |
| Age, mean (SD),y | 61.95 (12.14) | 63.76 (10.46) | .443 |
| Gender |  |  |  |
| Female | 11 (25.6) | 15 (30.0) | .636 |
| Male | 32 (74.4) | 35 (70.0) |  |
| **Cardiovascular risk factors** |  |  |  |
| Hypertension | 31 (72.1) | 40 (80.0) | .371 |
| Diabetes mellitus | 14 (32.6) | 23 (46.0) | .187 |
| Previous stroke | 13 (30.2) | 21 (42.0) | .240 |
| Atrial Fibrillation | 5 (11.6) | 2 (4.0) | .243 |
| Coronary artery disease | 5 (11.6) | 11 (22.0) | .186 |
| Cigarette smoking | 17 (40.5) | 24 (49.0) | .416 |
| Alcohol consumption | 15 (35.7) | 19 (38.8) | .763 |
| Antiplatelets | 11 (28.9) | 22 (44.0) | .149 |
| Intravenous Thrombolysis | 3 (7.1) | 2 (4.0) | .657 |
| NIHSS score at admission, median (IQR) | 6.0 (3.5- 9.5) | 7.0 (5.0- 8.75) | .682 |
| Transfer status |  |  |  |
| Direct admission | 20 (52.6) | 35 (70.0) | .096 |
| Transfer from another hospital | 18 (47.4) | 15 (30.0) |  |
| Time from LKW to arrival of treating hospital, median (IQR), h | 28.0 (25.0- 48.0) | 48.0 (27.25- 78.0) | .06 |
| Premorbid mRS |  |  |  |
| 0 | 36 (83.7) | 37 (74.0) | .163 |
| 1 | 6 (14.0) | 7 (14.0) |  |
| 2 | 0 (0.0) | 5 (10.0) |  |
| 3 | 1 (2.3) | 1 (2.0) |  |
| Occlusion site |  |  |  |
| M1 | 20 (46.5) | 19 (38.0) | .916 |
| M2 | 5 (11.6) | 5 (10.0) |  |
| ICA | 13 (30.2) | 18 (36.0) |  |
| Tandem | 4 (9.3) | 6 (12.0) |  |
| Others | 1 (2.3) | 2 (4.0) |  |
| ASPECT, median (IQR) | 8.0 (7.0- 9.0) | 8.0 (8.0- 9.0) | .378 |
| Ischemic Core Volume, median (IQR), mL | 7.60 (2.25- 16.70) | 2.70 (0.0- 31.97) | .195 |
| Mismatch Volume (IQR), ml | 48.90 (41.1- 75.5) | 109.10 (92.50- 113.03) | .104 |
| Tmax>6s (IQR), ml | 63.50 (51.72- 99.95) | 145.55 (92.50- 207.23) | .129 |
| Tmax>8s (IQR), ml | 30.0 (8.0- 61.0) | 73.25 (10.43-136.30) | .584 |
| Tmax>10s (IQR), ml | 16.0 (3.0- 33.0) | 23.05 (1.30- 89.05) | .784 |

### Abbreviations: ASPECT: Alberta Stroke Program Early CT Score; BMM: Best Medical Management; DDI: DAWN or DEFUSE 3 Ineligible; EVT: Endovascular Thrombectomy; ICA: Internal Carotid Artery; IQR: Interquartile Range; LKW: Last Known Well; mRS: Modified Rankin Scale; M1: Middle Cerebral Artery, Segment 1; M2: Middle Cerebral Artery, Segment 2; NIHSS: National Institutes of Health Stroke Scale; SD: Standard Deviation.

**Table S5. Standardized mean difference before and after each propensity score analysis**

|  | SMD/Raw | SMD/Matching | SMD/Weighting |
| --- | --- | --- | --- |
| Age | 0.216 | 0.001 | 0.018 |
| Sex | 0.101 | 0.014 | 0.006 |
| NIHSS | 0.231 | 0.019 | 0.062 |
| Pre-morbid mRS | 0.269 | 0.115 | 0.09 |
| Occlusion site | 0.12 | 0.069 | 0.076 |
| Time from LKW to arrival of treating hospital | 0.168 | 0.054 | 0.014 |

### Abbreviations: LKW: Last Known Well; mRS: Modified Rankin Scale; NIHSS: National Institutes of Health Stroke Scale; SMD: Standardized Mean Difference.
